# Predictors for day case surgery in shoulder arthroplasty: a study using the National Joint Registry and Hospital Episode Statistics for England

**DOI:** 10.1186/s13018-025-06440-5

**Published:** 2025-11-26

**Authors:** O. O’Malley, A. Davies, M. T. A. Sharabiani, A. Rangan, S. Sabharwal, P. Reilly

**Affiliations:** 1 Cutrale Perioperative and Ageing Group, Department of Bioengineering, Imperial College, London, UK; 2 Department of Primary Care and Public Health, School of Public Health, Imperial College, London, UK; 3 Hull York Medical School and Department of Health Sciences, University of York, Heslington, York YO10 5DD UK; 4 Department of Trauma and Orthopaedics, Imperial College Healthcare NHS Foundation Trust, London, UK

**Keywords:** Shoulder arthroplasty, Day case arthroplasty, Day case surgery, Day case shoulder arthroplasty

## Abstract

**Introduction:**

As shoulder arthroplasty volume increases in the UK, capacity challenges within a stretched health service mean that day case shoulder arthroplasty offers a value based solution. Patient selection for this is crucial and there is lack of evidence that informs guidance on patient selection for day case surgery. This study aims to use a large population reflective database, The National Joint Registry (NJR), to identify independent predictors for day case shoulder arthroplasty as well as develop a statistical tool that aids patient selection.

**Method:**

All shoulder arthroplasty procedures were requested from 1st April 2012 to 31st March 2022 from the NJR. These were then linked to the Hospital Episode Statistics for England (HES) to identify patient co-morbidities. A multivariable regression model was used to identify independent predictors of day case surgery. These predictors were then used to develop a clinical tool to predict likelihood of day case surgery with clinician inputted parameters.

**Results:**

There were 40,877 patients available for analysis. Younger age, being male having a lower ASA score, being operated on Monday-Thursday and having a Total Shoulder Arthroplasty or a Hemiarthroplasty rather than a Reverse Shoulder arthroplasty were significant predictors of having day case surgery. Having a diagnosis of dementia or paraplegia reduced the risk of having day case surgery. Using these predictive variables an excel prediction tool was developed with moderate predictive ability (AUC 0.65 GOF 0.82).

**Conclusion:**

This study has identified independent predictors for day case surgery and has developed a tool that can act as an adjunct for clinicians selecting patients for day case shoulder arthroplasty.

**Supplementary Information:**

The online version contains supplementary material available at 10.1186/s13018-025-06440-5.

## Background

Shoulder arthroplasty in the United Kingdom (UK) is increasing in use with a rise from 2764 cases in 2012 to 8221 in 2023 [1]. Traditionally shoulder arthroplasty has been performed as an inpatient with an average length of stay (LOS) reported as 1.31–2.8 days [2]. With the expansion of use as well as increasing waiting lists, day case or outpatient shoulder arthroplasty has been shown to be a safe and cost effective strategy that could reduce pressures on healthcare systems [2–4].

In order to complete successful day case surgery, appropriate patient selection and robust protocols are required. Although factors such as age, gender, low surgical volume and location have been shown to be predictors of prolonged LOS, there is limited literature on predictors on the likelihood of day case surgery as well as clinical tools to aid in identification of patients for day case surgery [5].

This study therefore aims to use a population based cohort, the National Joint Registry (NJR) for the UK to identify predictors for day case surgery as well as develop a tool that can be used clinically to identify patients that may be suitable for an outpatient pathway.

## Method

### Data source

Data was requested from the NJR for all shoulder arthroplasty procedures from 31st March 2012 to 1st April 2022. NJR data contains demographic, surgeon and procedural data. Patients were included if they had an elective shoulder arthroplasty and excluded if their procedure was for acute trauma. This database was linked to Hospital Episode Statistics (HES) for England which contains comorbidity data in the form of ICD-10 codes. Comorbidities contained in the Charlson Comorbidity Score (CCI) were individually identified and then a total CCI score calculated (Appendix 1) [6]. Prior to merging the number of arthroplasty procedures completed by each consultant on average per year in the registry was calculated as well as average number per year in the surgical unit. Length of stay was calculated from the primary procedure date in the NJR to the discharge date provided by HES.

### Statistical analysis

The analysis aimed to identify patients that could be targets for day case surgery. Aligning with previous studies in the literature, patients with 0 night stay (day case) and with a 1 night stay were compared with patients that had > 1 night stay with the aim to identify those patients that could be suitable for day case surgery [7]. Demographics between groups were compared using a chi squared test if categorial and T-Test or Wilcoxon rank-sum/Mann Whitney U dependant on the data skew in continuous outcomes. Significance was deemed at *p* < 0.050.

A univariable regression analysis was done for each demographic variable to assess for predictors for suitability for day case surgery. A multivariable regression analysis was then performed adjusting for all significant predictors to assess for independent factors that may predispose to possible suitability day surgery.

For development of the prediction tool independent factors were built into a regression model. Continuous variables were categorised to improve interpretability and facilitate clinical application. Age group was categorised as < 60, ≥ 60– < 70, ≥ 70– < 80 and ≥ 80. ASA was split into ASA 1–2 and 3–4. Unit cases per year were also split into quartile groups (lowest 25% <  = 17.00, 25–50% 17.01–26.00, 50–75% 26.01–37.74, 75% + 37.75). Pre-operative OSS was also categorised into quartiles as well as a missing group (lowest 25% <  = 10, 25–50% 11–16, 5–75% 17–22, 75% 22 +). Weekday operating was defined as operations taking place on Monday-Thursday and Weekend operating defined as taking place on a Friday to Sunday. During univariable analyses, an inverse relationship was observed between unit case volume and the likelihood of day case procedures, suggesting that higher-volume centres were less likely to perform day case surgery. This prompted further investigation into whether this finding was confounded by differences in patient case mix across centres. Specifically, we hypothesised that high-volume centres may manage a disproportionate number of older or more complex patients, leading to longer hospital stays. To explore this, an interaction term between age group and unit case volume was included. The interaction was retained based on model performance (likelihood ratio test between model *p* = 0.04) and clinical plausibility. The model was referenced to the average patient in the cohort: a 74-year-old female (age group 3) with an ASA of 2 in a medium volume unit (group 2) with the average preoperative function of the cohort (OSS group 2) reflecting the most common demographic and clinical profile in the study population. Area under curve (AUC) and Hosmer-Lewis Goodness of Fit test (GOF) was used to test the predictive model. The GOF for the above model was 0.82 with an AUC of 0.65. To attempt to improve model fit a Multivariable Fractional Polynomial analysis was tested to review non-linear associations, this however reduced GOF with a substantial increase in model complexity that would not be clinically useful and therefore the original model was used. The final model was internally validated using 200 bootstrap replications. The average optimism in model discrimination (AUC) was 0.005 (95% CI: –0.000 to 0.010; Standard Error (SE) 0.003; *p* = 0.07). This indicates minimal overfitting, with the optimism-corrected AUC estimated at approximately 0.66, supporting the model's internal validity.

To translate this model into a usable clinical tool, we constructed an Excel spreadsheet calculator. Each predictor is represented by a user-selectable dropdown menu, allowing clinicians to input individual patient characteristics. The underlying logistic regression formula, incorporating all regression coefficients (including interaction terms), was embedded directly in the spreadsheet using standard Excel functions (EXP, IF, AND). The tool calculates the predicted probability of same-day discharge in real-time upon data entry (supplementary material).

STATA statistics package (StataCorp. 2015. Stata Statistical Software: Release 14. College Station, TX: StataCorp LP) was used for the analysis. The STrengthening the Reporting of OBservational studies in Epidemiology (STROBE) guidelines were adhered to in this study [8].

## Results

**Fig. 1:**
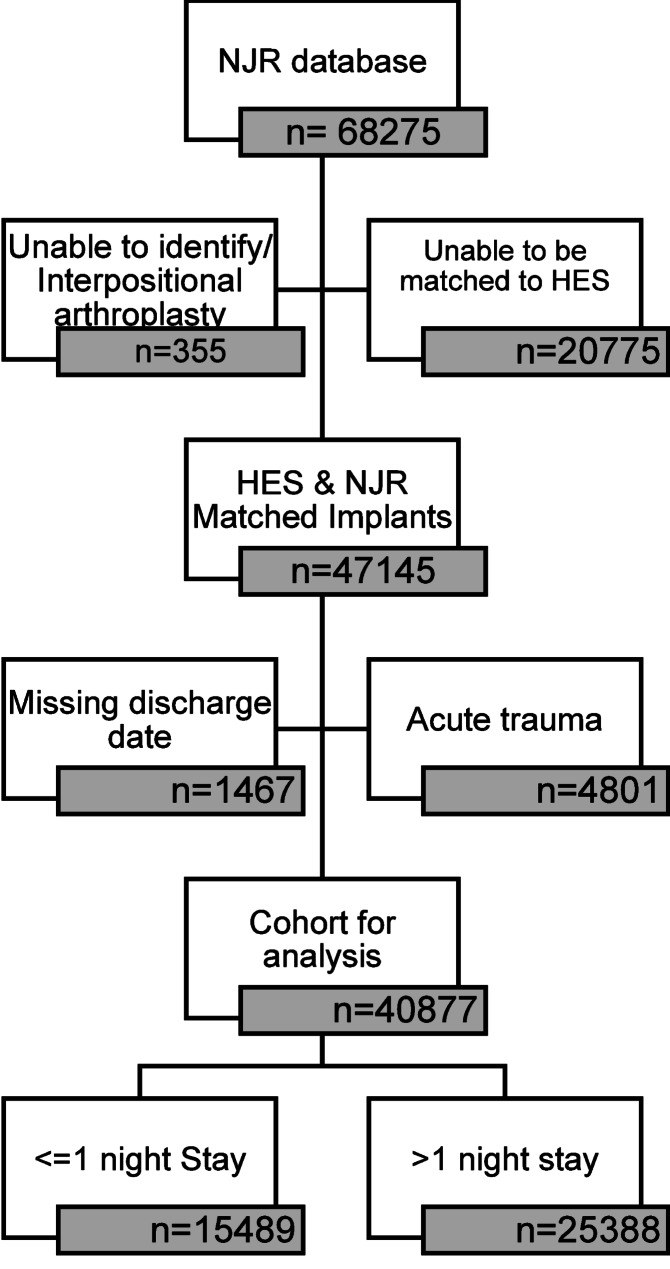
Study flow diagram

There were 40,877 elective shoulder arthroplasty procedures available for analysis between April 2012 and March 2022. Figure 1 shows the cohort for analysis and Table 1 shows the patients demographic data. 15,489 (37.89%) had a stay <  = 1 night.

**Table 1:** Demographics of those with a 1 night stay or less vs those with a greater than 1 night stay

Demographic	All implants			
	All (n = 40,877)	< = 1 (15,489)	> 1 (25,388)	*P* value
Age (IQR)	73.8 (67.4–79.1)	71.7 (65.3–77.1)	75.0 (68.9–80.2)	** < 0.001**
Gender (%) Female Male	28,437 (69.57) 12,440 (30.43)	9355 (60.40) 6134 (39.60)	19,082 (75.12) 6306 (24.84)	** < 0.001**
ASA (%) 1 2 3 4 + 5	2538 (6.21) 25,950 (64.38) 12,089 (29.57) 300 (0.74)	1227 (7.92) 10,549 (68.11) 3671 (23.70) 42 (0.27)	1311 (5.16) 15,401 (60.66) 8418 (33.16) 258 (1.02)	** < 0.001**
CCI total (IQR)	4 (3–5)	3 (3–5)	4 (3–5)	** < 0.001**
Cases per unit/year (IQR)	26.0 (17.0–37.75)	25.17 (16.42–35.75)	27.17 (17.25–45.33)	** < 0.001**
Cases per consultant/year (IQR)	17.73 (11.55–25.33)	17.73 (11.82–25.33)	17.64 (11.40–25.33)	0.681
Pre-operative OSS score (IQR)	16 (10–22)	17 (11–23)	15.00 (10–21)	** < 0.001**
Comorbidity (%) Previous myocardial infarction Congestive cardiac failure Peripheral vascular disease Cerebrovascular disease Dementia Chronic pulmonary disease Rheumatic disease Peptic ulcer disease Mild liver disease Diabetes without complications Diabetes with end organ damage Hemi or paraplegia Moderate or severe renal disease Malignancy except skin Lymphoma Leukaemia Moderate or severe liver disease Metastsis HIV/AIDS	2187 (5.35) 912 (2.23) 1570 (3.84) 2379 (5.82) 375 (0.92) 10,493 (25.67) 6027 (14.74) 1828 (4.47) 1152 (2.82) 6042 (14.78) 572 (1.40) 452 (1.11) 3719 (9.10) 3860 (9.44) 319 (0.78) 167 (0.41) 201 (0.49) 599 (1.47) 0 (0)	751 (4.85) 222 (1.43) 531 (3.43) 701 (4.63) 77 (0.50) 3659 (23.62) 2019 (13.04) 609 (3.93) 419 (2.71) 2101 (13.56) 173 (1.12) 106 (0.68) 1054 (6.80) 1358 (8.77) 114 (0.74) 67 (0.43) 67 (0.43) 208 (1.34) (0)	1436 (5.66) 690 (2.72) 1039 (4.09) 1678 (6.61) 298 (1.17) 6834 (26.92) 4008 (15.79) 1219 (4.80) 733 (2.89) 3941 (15.52) 399 (1.57) 346 (1.36) 2665 (10.50) 2502 (9.86) 205 (0.81) 100 (0.39) 134 (0.53) 391 (1.54) 0 (0)	** < 0.001** ** < 0.001** **0.001** ** < 0.001** ** < 0.001** ** < 0.001** ** < 0.001** ** < 0.001** 0.281 ** < 0.001** ** < 0.001** ** < 0.001** ** < 0.001** ** < 0.001** **0.011** 0.426 0.182 0.107 -
Day of week (%) Monday–Thursday Friday–Sunday	33,283 (81.42) 7594 (18.58)	12,868 (83.08) 2621 (16.92)	20,145 (80.41) 4973 (19.59)	** < 0.001**
Implant type RSA TSA HA	22,098 (54.06) 12,597 (30.82) 6182 (15.12)	7527 (48.60) 5285 (34.12) 2677 (17.28)	14,571 (57.39) 7312 (28.80) 3505 (13.81)	** < 0.001**

Bold values indicate equals statistically significant

Patients who had a LOS less than or equal to 1 night were younger, had a low CCI score, were more likely male, have a lower ASA score, higher pre-operative OSS scores and were done on a weekday (Monday-Thursday). RSA was the most likely implant to have a stay > 1 night (Table 1).

On multivariable regression modelling, age, gender, ASA score, unit caseload, having a diagnosis of dementia or paraplegia, weekend vs weekday operating and implant type were significant independent predictors for suitability for day case surgery (Table 2). Male patients were 64% (OR (95% CI) (1.64 (1.50–1.80)) more likely to have stays less than or equal to 1 night stay than female patients. Compared to RSA, TSA patients were 20% more likely and HA 17% more likely to have stays less than or equal to one night (OR (95% CI) 1.20 (1.10–1.32) & 1.17 (1.02–1.33)). Increasing age decreased the likelihood by 2% for every year increase in age and a diagnosis of hemiplegia/paraplegia or dementia both decreased the chances of LOS <  = 1 night by 42%. ASA score of 3 or greater also decreased likelihood of suitability for day case surgery. Being operated on at the weekend decreased the chances of LOS <  = 1 night by 21%.

**Table 2:** Univariable & multivariable regression analysis

Demographic	Univariate	*p*	Multivariate	*p*
Age	0.96 (0.96–0.97)	** < 0.001**	0.98 (0.97–0.99)	** < 0.001**
Gender Female Male	1 1.98 (1.90–2.07)	** < 0.001**	1 1.64 (1.50–1.80)	** < 0.001**
ASA 1 2 3 4	1 0.73 (0.67–0.79) 0.47 (0.43–0.51) 0.17 (0.12–0.24)	** < 0.001** ** < 0.001** ** < 0.001**	1 0.93 (0.79–1.11) 0.72 (0.59–0.86) 0.37 (0.19–0.70)	0.423 ** < 0.001** **0.002**
CCI total	0.86 (0.85–0.87)	** < 0.001**	0.99 (0.94–1.04)	0.652
Cases per unit/year	0.99 (0.99–0.99)	** < 0.001**	0.99 (0.98–0.99)	** < 0.001**
Cases per consultant/ year	0.99 (0.99–1.00)	** < 0.001**	1.00 (1.00–1.00)	0.848
Pre-operative OSS score	0.86 (0.85–0.87)	** < 0.001**	1.01 (1.01–1.02)	** < 0.001**
Comorbidity (%) Previous myocardial infarction Congestive cardiac failure Peripheral vascular disease Cerebrovascular disease Dementia Chronic pulmonary disease Rheumatic disease Peptic ulcer disease Mild liver disease Diabetes without complications Diabetes with end organ damage Hemi or paraplegia Moderate or severe renal disease Malignancy except skin Lymphoma Leukaemia Moderate or severe liver disease Metastsis	0.85 (0.78–0.93) 0.52 (0.45–0.61) 0.83 (0.75–0.92) 0.67 (0.61–0.73) 0.42 (0.33–0.55) 0.84 (0.80–0.88) 0.80 (0.76–0.85) 0.81 (0.73–0.90) 0.94 (0.83–1.06) 0.85 (0.81–0.90) 0.71 (0.59–0.85) 0.50 (0.40–0.62) 0.62 (0.58–0.67) 0.88 (0.82–0.94) 0.91 (0.72–1.15) 1.10 (0.81–1.50) 0.82 (0.61–1.10) 0.87 (0.73–1.03)	** < 0.001** ** < 0.001** **0.001** ** < 0.001** ** < 0.001** ** < 0.001** ** < 0.001** ** < 0.001** 0.281 ** < 0.001** ** < 0.001** ** < 0.001** ** < 0.001** ** < 0.001** 0.426 0.552 0.182 0.108	0.84 (0.69–1.03) 0.90 (0.65–1.24) 1.09 (0.86–1.37) 0.86 (0.70–1.06) 0.58 (0.35–0.98) 0.95 (0.85–1.06) 0.97 (0.85–1.10) 0.93 (0.75–1.15) 0.94 (0.82–1.08) 1.10 (0.75–1.60) 0.58 (0.36–0.95) 0.87 (0.72–1.04) 1.02 (0.83–1.25)	0.097 0.511 0.483 0.155 **0.041** 0.399 0.615 0.512 0.380 0.624 **0.032** 0.130 0.883
Day of week (%) Monday–Thursday Friday–Sunday	1 0.84 (0.79–0.88)	** < 0.001**	1 0.79 (0.71–0.88)	** < 0.001**
Implant type RSA TSA HA	1 1.40 (1.34–1.46) 1.48 (1.40–1.57)	** < 0.001** ** < 0.001**	1 1.20 (1.10–1.32) 1.17 (1.02–1.33)	** < 0.001** **0.022**

Bold values indicate equals statistically significant

When these significant predictors were modelled on the average patient (Female, aged 74 in a medium volume unit with the average pre-operative function) (Table 3). After adjusting for covariates, a significant interaction was observed between age group and unit case volume (*p* < 0.001). Patients under 60 had significantly higher odds of undergoing day case procedures in low- and mid-volume centres (e.g. OR = 1.59 (95% CI 1.37–1.84) in Q1 & OR = 1.78 (95% CI 1.54–12.05) in Q3), but had reduced odds in high-volume centres (Q4: OR = 0.79 (95% CI 0.69–0.90)). To note patients under 60 were most commonly done in a high volume centre (Q4). This trend was also seen in the 60–70 age group, though the attenuation was less pronounced. Conversely, patients over 80 had substantially reduced odds of day case surgery across all unit volumes (Q4: OR = 0.40 (95% CI 0.35–0.45)), suggesting as seen in the original multivariable model that increasing age and unit volume are independently associated with decreased likelihood of day case surgery.

**Table 3:** Prediction regression model

Demographic	Number of patients	OR (95% CI)	*P* value
Age × unit cases < 60 × Q1 (Low volume unit) < 60 × Q2 < 60 × Q3 < 60 × Q4 (High volume unit) ≥ 60–< 70 × Q1 ≥ 60–< 70 × Q2 ≥ 60– < 70 × Q3 ≥ 60-–< 70 × Q4 ≥ 70–< 80 × Q1 ≥ 70–< 80 × Q2 ≥ 70–< 80 × Q3 ≥ 70–< 80 × Q4 ≥ 80 × Q1 ≥ 80 × Q2 ≥ 80 × Q3 ≥ 80 × Q4	930 (22.31) 1028 (24.66) 997 (23.91) 1214 (29.12) 2457 (25.81) 2349 (26.77) 2315 (24.31) 2390 (25.10) 4821 (26.24) 4553 (24.78) 4573 (24.89) 4428 (24.10) 2120 (24.06) 2178 (11.85) 2366 (26.85) 2148 (24.38)	1.59 (1.37–1.84) 1.36 (1.18–1.57) 1.78 (1.54–12.05) 0.79 (0.69–0.90) 1.41 (0.69–0.90) 1.28 (1.15–1.41) 1.38 (1.24–1.52) 0.88 (0.80–0.98) 1.09 (1.00–1.19) 1 (reference group) 1.06 (0.98–1.16) 0.62 (0.57–0.68) 0.60 (0.53–0.67) 0.61 (0.54–0.68) 0.63 (0.56–0.70) 0.40 (0.35–0.45)	** < 0.001** ** < 0.001** ** < 0.001** ** < 0.001** ** < 0.001** ** < 0.001** ** < 0.001** **0.020** **0.044** 0.151 ** < 0.001** ** < 0.001** ** < 0.001** ** < 0.001** ** < 0.001**
Gender Female Male	28,437 (69.57) 12,440 (30.43)	1 1.79 (1.71–1.13)	** < 0.001**
ASA 1–2 3 +	28,488 (69.69) 12,389 (30.31)	1 0.68 (0.65–0.71)	** < 0.001**
Pre-Operative OSS Missing Q1 low Q2 Q3 Q4 high	30,227 (73.95) 2706 (6.62) 3047 (7.45) 2459 (6.02) 2438 (5.96)	1.04 (0.96–1.13) 0.87 (0.78–0.98) 1 1.03 (0.92–1.16) 1.07 (0.95–1.19)	0.299 **0.018** 0.588 0.273
Dementia	375 (0.92)	0.58 (0.44–0.74)	** < 0.001**
Hemi or paraplegia	452 (1.11)	0.52 (0.42–0.65)	** < 0.001**
Day of week (%) Monday–Thursday Friday–Sunday	33,283 (81.42) 7594 (18.58)	1 0.80 (0.75–0.84)	** < 0.001**
Implant type RSA TSA HA	22,098 (54.06) 12,597 (30.82) 6182 (15.12)	1 1.12 (1.07–1.18) 1.16 (1.09–1.24)	** < 0.001** ** < 0.001**

Bold values indicate equals statistically significant

## Discussion

In the USA & Canada various studies have shown that day-case shoulder arthroplasty can be achieved safely with high patient satisfaction [3, 9, 10]. Despite this, outpatient shoulder arthroplasty in the UK is rare with a recent study showing 1.24% of RSA procedures being completed as a day case [4]. Hip and knee arthroplasty research has demonstrated that careful patient selection is crucial in developing a successful pathway, however the evidence to inform on this for shoulder arthroplasty in the UK is limited to a select group of single centre studies [2, 11, 12]. Allen et al. using a literature search and discussion with a multidisciplinary team, developed a five phase pathway for day case patients [12]. The primary phase involved careful patient selection. Patients had to be under 75, reflective of a further UK study that saw no complications or readmission in 21 RSA day case procedures in a cohort with a mean age of 74, have no history of cardiopulmonary or thrombotic disease/ ASA < 3, a BMI under 35, live within 1 h of the hospital, have someone available to stay overnight with them after the surgery and not be reliant pre-operatively on opioids [12]. The development of this pathway resulted in four successful day case procedures and on retrospective review they identified that 22% of their cohort would have been suitable for day case surgery on this pathway however the pathway has not been validated due to the small sample size [12]. The UK study of 21 RSA patients also had similar criteria for day case surgery: patients were excluded if they lived alone and someone was not present overnight following the procedure, if patients had ongoing medical issues including sleep apnoea, were on anticoagulation or if they had neurologic compromise preventing independent mobility [2].

This large population based cohort study has identified both patient and surgical factors that can independently predict likelihood of day case surgery. The literature is clear that prolonged length of stay is associated with increasing age and a systematic review looking at a pooled 7162 outpatient shoulder arthroplasties found the average age of patients to be 66.6 years [5, 9]. Our study has shown with every year of increasing age there is reduction of 2% in the likelihood of day case surgery [5]. In our predictive tool age was categorised into groups for ease of clinical use and the effect of age was influenced by unit volume. In high volume units across all age groups there was a reduced likelihood of day case surgery. It is established that higher volume surgeons are less likely to have complications such as revision and re-operations in shoulder arthroplasty and in hip and knee and elbow arthroplasty, higher unit volume has been associated with better clinical outcomes [13–15]. We hypothesise that our finding of higher volume units having a lower chance of day cases may be due to more complex procedures being completed in these units but also dedicated elective centres may not have the same bed pressures traditional inpatient units. However, this cannot be proven from our study and warrants further investigation. In low to medium volume units, younger age was associated with higher likelihood of day case surgery however in those > 80 age in all unit sizes there was a lower chance of day case surgery.

Co-morbidities such as congestive cardiac failure, renal failure and pulmonary disease have previously been shown to be associated with prolonged length of stay however dementia and hemi or paraplegia were the only co-morbidities in this study to be independently associated with reduced likelihood of day case surgery [5, 16]. The study by Tansey et al. however did exclude patients that had neurologic compromise preventing independent mobility from day case surgery of which both dementia and hemi or paraplegia would be included in [2]. In agreement with our study a lower ASA score and CCI was seen in day case vs inpatient shoulder arthroplasty in a systematic review [9]. In our study an ASA of 3 + conferred a 32% reduction in likelihood of day case surgery and this was also an exclusion criteria for the day case pathway in the study by Allen et al. [12].

In hip & knee arthroplasty it has been shown that day of the week can affect length of stay however other publications have rebutted this [17, 18]. Our study showed that being operated on Friday-Sunday reduced the likelihood of day case surgery by 16%. This may be due to staffing availability or lack of day case centres that operate over weekends. Although this may not directly affect patient selection, it does suggest that staffing levels or expertise on certain days of the week could increase the success of implementing day case procedures on those specific days.

Finally implant type was shown to affect likelihood of day case surgery. Using RSA as a control both HA and TSA had a 40 and 48% increased likelihood of day case surgery compared to RSA. TSA & HA patients are on average younger than RSA patients and inherently therefore less comorbid which may contribute to this finding [1].

This study involves a large population reflective cohort and has provided insights into patient selection for day case surgery as well as producing a useable tool for implementation. There are however limitations. The predictive tool developed enables real time prediction of day case probability based on patient as well as local factors. An AUC of 0.65 indicates moderate discrimination at best. This limits the tool’s standalone clinical usefulness although it may be better suited as an adjunct rather than a sole decision-making guide. Existing research has shown that social factors and mobility levels also have a strong influence on patients successful selection of shoulder day case arthroplasty patients. This data is lacking from NJR and HES data sets and therefore these factors could not be included in this analysis. It would be prudent to consider them in patient selection for day case surgery and future work using national datasets could potentially be supplemented with data from primary care. The sample size was also reduced due to merging of databases however the cohort remains large. Finally, the PROMs data had a large amount of missing data, this was addressed by adding a missing field to address the lack of pre-operative data in some patients. Despite these limitations, this is a large population representative dataset with real world relevance. The use of the prediction tool in clinical practice or development of day case pathways based on the predictors identified in this study to complete day case practice should be targets for future research to identify the outcomes of these selected day case patients.

## Conclusion

Overall, this study has shown that age, gender, ASA score, unit caseload, having a diagnosis of dementia or paraplegia, day of the week and implant type are independent predictors for day case surgery. Along with identification of these factors, a clinical tool has been developed that can act as adjunct to aid in the appropriate selection of patients for day case shoulder arthroplasty.

## Supplementary Information

Below is the link to the electronic supplementary material.

Supplementary file1 (DOCX 15 kb)

Supplementary file2 (XLSX 10 kb)

## Data Availability

The data in this study was requested from the National Joint registry. The data is not publicly available and cannot be shared by the corresponding author.

## Abbreviations

NJR: National Joint Registry
HES: Hospital episode statistics
ICD-10: International classification of diseases version 10
AUC: Area under curve
GOF: Goodness of fit
LOS: Length of stay
CCI: Charlson co-morbidity index
OSS: Oxford shoulder score
SE: Standard error
ASA: American society of anesthesiologists physical status score
RSA: Reverse shoulder arthroplasty
TSA: Total shoulder arthroplasty
HA: Hemiarthroplasty
