# Supplementary material for: Predictors for day case surgery in shoulder arthroplasty: a study using the National Joint Registry and Hospital Episode Statistics for England
Source: J Orthop Surg Res. 2025 Nov 26;20:1042. doi: 10.1186/s13018-025-06440-5 (PMC12659241; doi:10.1186/s13018-025-06440-5)
Supplement: Supplementary file 1 — Supplementary file1 (DOCX 15 kb) [file 13018_2025_6440_MOESM1_ESM.docx]

Appendix 1

ICD-10 Codes for co-morbidities

| Comorbidity | ICD – 10 code |
| --- | --- |
| Myocardial Infarction | I21, I22, I252 |
| Congestive Cardiac Failure | I099, I110, I130, I132, I255, I420, I425-I429, I43, I50, P290 |
| Peripheral Vascular Disease | I70, I71, I73.1, I73.8, I73.9, I77.1, I79.0, I79.2, K55.1, K55.8, K55.9, Z95.8, Z95.9 |
| Cerebrovascular Disease | G45, G46, H34, I60-I69 |
| Dementia | F00-F03, F051, G30, G311 |
| Chronic Pulmonary Disease | I278, I279, J40-J47, J60-J67, J684, J701, J703 |
| Rheumatic disease | M05, M06, M120, M315, M32-M34, M351, M353, M360 |
| Peptic Ulcer Disease | K25-K28 |
| Mild Liver Disease | B18, K700-K703, K709 , K713 - K715, K717, K73, K74, K752, K754, K758, K759, K760- K764, K768, K769, Z944 |
| Diabetes without chronic complications | E100, E101, E109, E110, E111, E119, E120, E121, E129, E130, E131, E139, E140, E141, E149 |
| Diabetes with end organ damage | E102- E105, E107, E112- E115, E117, E122-E125, E127, E132- E135, E137, E142- E145, E147, N083 |
| Hemi or paraplegia | G041, G114, G800, G801, G802, G81, G82, G830, G839 |
| Moderate or severe renal disease | I120, I131, I132, N03 , N012 -N017, N032-N037, N042-N047, N052-N057, N072-N077, N183-N185, N19, N250, N280, N290, Z490-Z492, Z992 |
| Malignancy except skin | C00–C26, C30–C34, C37–C41, C43, C45–C58, C60–C76, |
| Lymphoma | C81-C88, C90 |
| Leukemia | C91-C96 |
| Moderate or Severe liver disease | I85, I864, I982, I983, K704, K711, K713, K721, K729, K762, K763, K765-K767 |
| Mets | C77-C80 |
| HIV/Aids | B20-B24 |
